# Insertable cardiac monitor malfunction secondary to alexandrite laser procedure

**DOI:** 10.1016/j.jdcr.2024.04.044

**Published:** 2024-05-10

**Authors:** Cameron M.B. Zachary, Andrew Creadore, Solomiya Grushchak, Christopher B. Zachary

**Affiliations:** Department of Dermatology, University of California, Irvine, California

**Keywords:** 755 nm, alexandrite, error, ICM, ILR, implantable loop recorder, insertable cardiac monitor, laser, malfunction, reset, safety

## Case report

An 85-year-old woman with a history of atrial fibrillation status post ablation and placement of Medtronic REVEAL LINQ insertable cardiac monitor (ICM) presented to our clinic for treatment of solar lentigines and flat seborrheic keratoses on the chest with a Lutronic CLARITY II Alexandrite 755 nm laser. Two passes were performed: first pass with a 16 mm spot size, 5 ms pulse duration, 5.5 J/cm^2^ energy, 0/0/20 cooling, and second pass with an increase in pulse duration to 12 ms. She tolerated the procedure well without any complaints. Shortly after, the patient’s cardiologist informed her that he received an alert that her ICM had been *reset*.

## Discussion

Insertable cardiac monitors (aka implantable loop recorders) are electrocardiograms (ECGs) that are implanted by a cardiologist subcutaneously in the chest overlying the heart and functions to detect underlying arrythmias.^1^ They are placed ∼8 mm below the epidermis and are protected by a metal alloy casing that holds a battery, which powers the device for 3 to 4 years.^1^ An ICM continuously records the electrical signals from the heart and has various parameters tailoring the device to a specific patient.^1^^,^^2^ Parameter examples include setting cutoffs for minimum and maximum beats per minute and programming sensitivities to specific arrythmias. ECG data are then wirelessly transmitted to a separate at-home Patient Assistance device and, ultimately, sent to a central data center for analysis.

A “device reset” is a safety feature to prevent severe damage of the cardiac monitor, however, a reset itself can result in the loss of stored data and changes in the settings of programmed parameters.^2^ The loss of ECG data containing dangerous arrythmias has clear and adverse implications for the patient. Altered parameters may lead to aberrant signaling to central data centers causing confusion and diverting health care resources. Furthermore, patients with older models need to have reset ICMs rebooted by technicians at an outpatient cardiology clinic requiring an in-person visit. Newer devices can be rebooted remotely, which our patient was equipped with, yet a reboot error required our patient to visit in-person, nonetheless.

An ICM operates via electrical impulses traveling along a circuit between electrodes. These circuits can be temperature sensitive, where alterations in temperature can lead to changes in resistances across the circuit, resulting in improper signaling and malfunction of the underlying algorithm. Our leading theory for the ICM reset postulates that 755 nm scattered light reflected off the titanium nitride electrodes located inside the ICM, leading to alterations in temperature, compromising the algorithm and triggering a device reset. A 755 nm laser penetrates ∼2 to 3 mm below the epidermis; however, nontherapeutic scattered photons may be able to reach the implantable loop recorder at ∼8 mm, particularly with a spot size of 16 mm (James Muze, Chief Technology Officer of Lutronic, written communication, March 2024).

Literature review did not reveal a single case of ICM device reset, or any type of interference, with alexandrite 755 nm laser devices or any other laser or energy-based dermatologic devices. Although commonly encountered electromagnetic devices such as cellular phones and metal detectors have failed to demonstrate interference of an ICM, interference has been demonstrated *in vivo* from radiofrequency electronic article surveillance systems, better known as retail theft devices, via electromagnetic interference.^3^ This interference is caused by proximity of electromagnetic fields that are naturally created by any device that uses electricity or transmits wireless signals. Laser-based devices need to meet specific thresholds of electromagnetic compatibility set by the standards of the International Electrotechnical Commission 60601 to be approved by the Food and Drug Administration, thus electromagnetic interference from our laser device unlikely caused the reset. Apart from device reset, other forms of interference of ICMs, such as false positive arrhythmia recordings, have been reported with transcutaneous electrical nerve stimulation^4^ and electrocautery.^5^ Based on available published data, device malfunctions such as these appear to be exceedingly rare, with most reported adverse outcomes of these devices relating to complications of implantation (ie, infection, pain, and scar formation).^6^

To prevent future occurrences, we recommended providers screen for ICMs during initial consultation for laser procedures. Patients with ICMs seeking laser treatment for lesions on the chest should be made aware of the risk of a device reset, as there is no “safety mode” that can temporarily shut off the ICM during procedures.^2^ To our knowledge, there is no recommended safe distance between a laser-based device used in proximity to an ICM. Manufacturers of ICMs have made recommendations for household and office appliances that give off elevated levels of electromagnetic interference. However, as laser-based devices are built to be electromagnetically compatible, these ranges do not apply. A laser-based device should never be pointed and operated toward any implantable cardiac device. When spot-treating lesions close to cardiac monitors, we believe a conservative spot size of 3 or 4 mm may help prevent scattered photons from reaching the ICM (James Muze, written communication, March 2024).

This case highlights that iatrogenic ICM device resets can lead to loss of ECG data that may reveal potentially fatal arrythmias and wasting of health care resources. Physicians using an alexandrite 755 nm device should be aware of this complication and screen patients for ICM before proceeding with treatment. Future complications may be avoided with thorough investigation into interference between different lasers with common implantable devices.

## Conflicts of interest

None disclosed.
